# Œdème aigue du poumon en rapport avec un myxome enclavé dans la valve mitrale

**DOI:** 10.11604/pamj.2016.23.205.8360

**Published:** 2016-04-20

**Authors:** Ali Derkaoui, Mohammed Khatouf

**Affiliations:** 1 CHU Hassan II, Université sidi Mohammed Benabdellah, Service de Réanimation Polyvalente, Fès, Maroc

**Keywords:** Myxome, œdème aiguë du poumon, chirurgie, Myxoma, acute pulmonary edema, surgery

## Image en médecine

Les myxomes de l'oreillette gauche sont des tumeurs bénignes pouvant engager le pronostic vital en rapport avec leurs localisations. Nous rapportons l'observation d'une patiente âgée de 70 ans, admise dans notre formation pour la prise en charge d'une détresse respiratoire en rapport avec un œdème aigue du poumon hémodynamique. L échocardiographie a objectivé un myxome enclavé dans la valve mitrale associé à une dilatation de l'oreillette gauche et une hypertension artérielle pulmonaire. La patiente a été opérée en urgence sous circulation extracorporelle pour résection tumorale.

**Figure 1:**
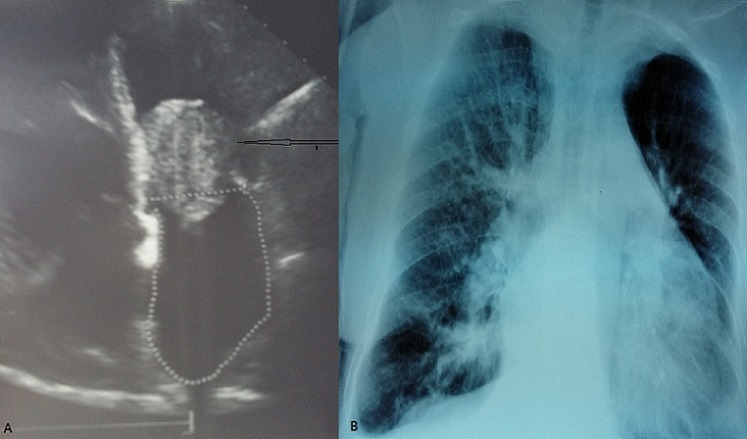
A) échocardiographie coupe 4 cavités montrant un myxome envalé dans la valve mitrale (tête de flèche); B) radiographie pulmonaire de face montrant un infiltrat alvéolo-interstitiel bilatéral faisant évoqué un œdème aiguë du poumon

